# Preoperative electrolyte-based metabolic biomarkers for prognostic assessment in gastric cancer: a multicenter study

**DOI:** 10.3389/fendo.2026.1852688

**Published:** 2026-06-11

**Authors:** Weihao Kong, Jiawen Wang, Xuqin Wu, Kangjie Zhang, Xingyu Wang, Jianlin Zhang

**Affiliations:** 1 Department of Emergency Surgery, The First Affiliated Hospital of Anhui Medical University, Hefei, China; 2 West China Clinical Medical College, West China Hospital, Sichuan University, Chengdu, Sichuan, China; 3 Medical Records Management Department, Lujiang People’s Hospital of Anhui Province, Hefei, China

**Keywords:** electrolyte score, gastric cancer, metabolic biomarkers, nomogram, prognostic stratification

## Abstract

**Background:**

Gastric cancer remains a major cause of cancer-related mortality worldwide, with substantial heterogeneity in patient outcomes despite advances in treatment. Clinically accessible biomarkers are still needed to improve prognostic stratification. Serum electrolytes, as routine circulating parameters, may reflect systemic metabolic status, host physiological adaptation, and tumor-related stress. This study aimed to develop and externally validate an electrolyte-based metabolic biomarker score for prognostic assessment in gastric cancer.

**Methods:**

This retrospective multicenter study included 1, 371 patients with gastric cancer from two independent cohorts: a training cohort (n = 1, 085) and an external validation cohort (n = 286). Seven preoperative serum electrolytes were analyzed. Univariate and multivariate Cox regression analyses were used in the training cohort to identify independent prognostic electrolytes and construct an Electrolyte Score. Patients were stratified into high- and low-risk groups according to the median score in the training cohort, and the same cutoff was applied to the validation cohort. Kaplan-Meier analysis, time-dependent ROC analysis, calibration curves, and decision curve analysis were used to evaluate prognostic performance and clinical utility.

**Results:**

Sodium and calcium were identified as independent prognostic factors for overall survival and incorporated into the Electrolyte Score. Higher sodium and calcium levels were associated with better survival. Although the Electrolyte Score alone had limited discriminatory ability, it was able to stratify patients into prognostically distinct groups in both cohorts. A high Electrolyte Score was associated with worse overall survival (training cohort, P < 0.001; validation cohort, P = 0.004). Multivariate analysis confirmed the score as an independent prognostic factor. Importantly, a nomogram integrating the Electrolyte Score with age, TNM stage, and histological differentiation improved individualized prognostic prediction, demonstrating potential clinical utility for risk stratification and guiding patient management.

**Conclusions:**

A preoperative electrolyte-based score derived from sodium and calcium provided independent prognostic information in gastric cancer. While its predictive value alone is limited, its combination with clinicopathological variables in a nomogram enhances individualized risk stratification, offering a practical tool to complement conventional prognostic assessment in clinical practice.

## Introduction

Gastric cancer is among the most prevalent malignant neoplasms worldwide and remains a major contributor to cancer-related deaths. Although considerable progress has been made in surgical management, chemotherapy, and targeted treatment, the overall long-term outcomes of patients with gastric cancer are still unsatisfactory, especially in those diagnosed at an advanced stage (1–6). Currently, prognostic evaluation mainly relies on clinicopathological factors such as tumor-node-metastasis (TNM) staging and histological differentiation (7–9). However, patients with similar clinical stages often exhibit markedly heterogeneous outcomes, highlighting the need for more reliable and easily accessible prognostic biomarkers.

Increasing evidence has demonstrated that metabolic reprogramming and alterations in the tumor microenvironment play critical roles in the progression of gastric cancer (10, 11). Electrolytes, including potassium, sodium, chloride, bicarbonate, calcium, magnesium, and phosphate, are essential regulators of cellular metabolism, acid–base balance, and intracellular signaling (12–16). Dysregulation of these ions can influence tumor cell proliferation, apoptosis, invasion, and immune responses within the tumor microenvironment (17–21). For instance, calcium signaling is closely associated with cancer cell migration and metastasis, while sodium and bicarbonate are involved in maintaining pH homeostasis, which may facilitate tumor growth and resistance to therapy (22–26). Notably, these electrolyte parameters are routinely measured in clinical practice, making them readily accessible, cost-effective, and routinely accessible biomarkers with potential prognostic value.

Although individual electrolyte abnormalities have been reported to be associated with cancer progression and patient outcomes, most existing studies have focused on single parameters rather than evaluating their combined effects. A comprehensive assessment integrating multiple electrolyte indicators may better reflect the complex metabolic status and tumor microenvironment in gastric cancer patients. However, to date, there is a lack of systematic approaches to construct and validate an electrolyte-based prognostic model in this population.

In the present study, we systematically analyzed seven common serum electrolytes, including potassium, sodium, chloride, bicarbonate, calcium, magnesium, and phosphate, in patients with gastric cancer. Through univariate and multivariate Cox regression analyses, we identified key prognostic electrolytes and established a novel composite index termed the Electrolyte Score. We further evaluated its prognostic value in both a training cohort and an external validation cohort. In addition, we integrated the Electrolyte Score with clinicopathological characteristics to develop and validate a predictive nomogram for individualized survival assessment.

## Materials and methods

### Study population

This retrospective, multicenter study included patients with gastric cancer from two independent cohorts. The training cohort consisted of patients treated at the First Affiliated Hospital of Anhui Medical University between January 2012 and December 2014. The external validation cohort comprised patients from Lujiang County Hospital of Anhui Province, collected between December 2016 and October 2018. Eligible patients were adults (≥18 years) with histopathologically confirmed gastric cancer treated at the participating centers within the defined study periods. Patients were excluded if they had: 1) incomplete clinical records; 2) missing preoperative serum electrolyte measurements; 3) incomplete follow-up information; or 4) concurrent severe comorbidities (e.g., advanced hepatic or renal failure) that could influence metabolic or electrolyte status. Specifically, 58 patients (≈5.1%) in the training cohort and 25 patients (≈8.0%) in the validation cohort were excluded due to missing data. Baseline clinicopathological characteristics, including age, gender, TNM stage, and histological differentiation, were collected for further analysis. All patients were followed up regularly after treatment, and overall survival was defined as the time from diagnosis to death from any cause or the last follow-up. This study was conducted in accordance with the Declaration of Helsinki and was approved by the institutional ethics committees of the participating centers.

### Data collection

Clinical and laboratory data were retrospectively collected from electronic medical records at each participating center. All serum electrolyte measurements were obtained prior to surgical treatment. The electrolyte parameters included potassium (K), sodium (Na), chloride (Cl), bicarbonate (HCO_3_^-^), calcium (Ca), magnesium (Mg), and phosphate (P). Baseline clinicopathological characteristics, including age, gender, TNM stage, and histological differentiation, were also collected for subsequent analyses. Laboratory measurements were performed according to standard procedures at each institution. Although assays were conducted at different centers, all electrolyte parameters were reported using consistent units, ensuring comparability across cohorts.

### Statistical analysis

All statistical analyses were carried out using R software (version 4.3.2) and SPSS software (version 25; IBM Corp., Armonk, NY, USA). Statistical significance was defined as a two-sided P value of less than 0.05. In the training cohort, the prognostic associations of seven serum electrolyte variables, including potassium (K), sodium (Na), chloride (Cl), bicarbonate (HCO_3_^-^), calcium (Ca), magnesium (Mg), and phosphate (P), were first assessed by univariate Cox proportional hazards regression analysis. Electrolytes with P values below 0.05 in the univariate analysis were then entered into a multivariate Cox regression model to determine independent prognostic factors. An Electrolyte Score was subsequently established according to the regression coefficients obtained from the multivariate model, and the same formula was applied to calculate the score for each patient in both the training and validation cohorts. The median value of the Electrolyte Score in the training cohort was selected as the predefined cut-off because no established clinical threshold is currently available for this newly developed biomarker. The same cut-off was applied unchanged to the validation cohort to avoid overfitting and data leakage. Survival curves were estimated using the Kaplan-Meier method, and differences between groups were evaluated with the log-rank test. To determine whether the Electrolyte Score was an independent prognostic factor, univariate and multivariate Cox regression analyses were performed incorporating clinicopathological variables, including age, gender, TNM stage, histological differentiation, and the Electrolyte Score. A prognostic nomogram was developed based on the independent prognostic factors identified in the multivariate Cox analysis of the training cohort. The predictive performance of the nomogram was evaluated using time-dependent receiver operating characteristic (ROC) curves at 1-, 2-, 3-, 4-, and 5-year time points. Calibration curves were plotted to assess the agreement between predicted and observed survival probabilities. Decision curve analysis (DCA) was conducted to evaluate the clinical utility of the model by quantifying net benefits at different threshold probabilities. The performance of the nomogram was further validated in the external validation cohort using the same methods.

## Results

### Baseline characteristics

This study included 1, 371 patients with gastric cancer, of whom 1, 085 were assigned to the training cohort and 286 to the validation cohort. The baseline clinicopathological characteristics of the two cohorts are shown in **Table 1**. In the training cohort, 470 patients were aged ≥65 years, while 615 were younger than 65 years. In contrast, the validation cohort included 200 elderly patients (≥65 years). The gender distribution was comparable between the two cohorts (P = 0.533), with males accounting for the majority of patients in both groups. Significant differences were observed in tumor characteristics between the two cohorts. Patients in the training cohort were more likely to present with advanced TNM stage (P < 0.001) and poorly differentiated tumors (P < 0.001). In addition, survival status differed between the cohorts (P = 0.014), and the validation cohort had a longer mean follow-up time compared to the training cohort (1786 ± 1038 *vs*. 1541 ± 1005 days, P < 0.001). Overall, several baseline characteristics, including age, TNM stage, differentiation, and follow-up time, showed significant differences between the training and validation cohorts.

**Table 1:** Baseline clinicopathological characteristics of gastric cancer patients in the training and validation cohorts.

Characteristics	Training cohort	Validation cohort	P value
n	1085	286	
Age, n (%)			< 0.001
≥65	470	200	
<65	615	86	
Gender, n (%)			0.533
female	293	72	
male	792	214	
TNM stage, n (%)			< 0.001
I+II	388	140	
III+IV	697	146	
Differentiation, n (%)			< 0.001
Moderately-Well	675	90	
Poorly	410	196	
Event, n (%)			0.161
alive	462	135	
dead	623	151	
time, mean ± sd	1541 ± 1005	1786 ± 1038	< 0.001

### Identification of prognostic electrolytes

**Figure 1:**
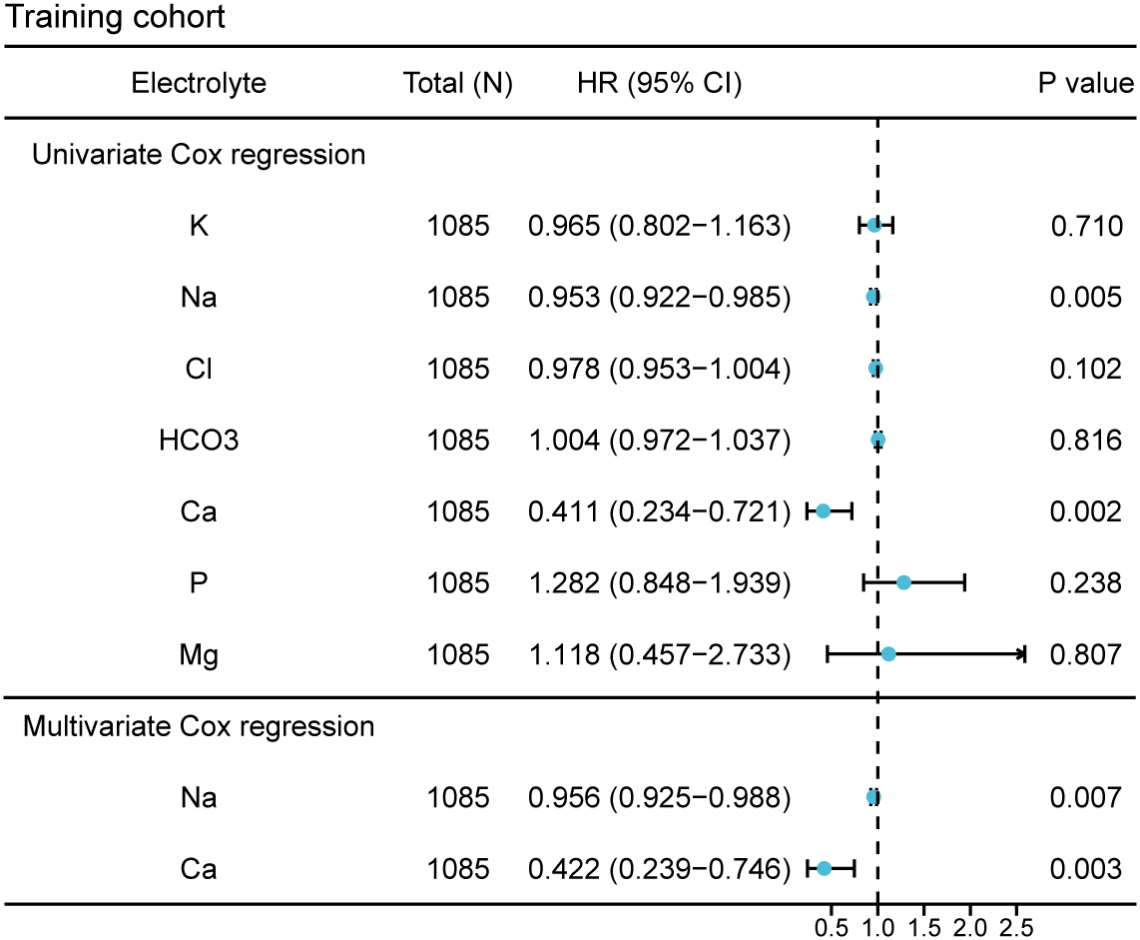
Forest plot of multivariate Cox regression analysis in the training cohort showing the independent prognostic value of serum sodium (Na) and calcium (Ca) for overall survival in gastric cancer patients.

Univariate Cox regression analysis performed in the training cohort showed that, among the seven serum electrolytes evaluated, sodium (Na) and calcium (Ca) were significantly correlated with overall survival (both P < 0.05). These two variables were subsequently incorporated into a multivariate Cox regression model to determine whether they independently predicted prognosis. As shown in **Figure 1**, both Na and Ca remained independent predictors of survival. Higher Na levels were associated with improved prognosis (hazard ratio [HR] = 0.956, 95% confidence interval [CI]: 0.925–0.988, P = 0.007), while higher Ca levels were similarly protective (HR = 0.422, 95% CI: 0.239–0.746, P = 0.003). These findings suggest that both Na and Ca are independent prognostic electrolytes in gastric cancer, supporting their inclusion in the construction of a composite Electrolyte Score.

### Construction of the electrolyte score

Based on the coefficients derived from the multivariate Cox regression model, an Electrolyte Score was constructed for each patient as follows: Electrolyte Score = (−0.0454×Na) + (−0.8628×Ca). Because both Na and Ca were protective factors with negative coefficients, higher Electrolyte Score values mainly reflected lower Na and/or Ca levels and were therefore associated with poorer prognosis. The time-dependent ROC analysis of the Electrolyte Score alone yielded 1-, 3-, and 5-year AUC values of 0.564, 0.564, and 0.554 in the training cohort, and 0.600, 0.550, and 0.588 in the validation cohort (**Supplementary Figures 1A, B**). Patients were divided into high- and low-risk groups based on the median Electrolyte Score in the training cohort, and this cutoff was then used for stratification in the validation cohort. Kaplan–Meier survival analysis demonstrated that patients in the high-score group had significantly poorer overall survival than those in the low-score group in both the training cohort (P < 0.001) and the validation cohort (P = 0.004). (**Supplementary Figures 1C, D**). These findings suggest that the Electrolyte Score can stratify patients into distinct prognostic groups, although its discriminative performance alone was limited.

### Subgroup survival analysis according to TNM stage and histological differentiation

To assess whether the prognostic performance of the Electrolyte Score remained consistent across clinically relevant subgroups, Kaplan–Meier survival analyses were conducted after stratifying patients by TNM stage and histological differentiation in both cohorts. In the training cohort, the Electrolyte Score did not significantly distinguish overall survival among patients with TNM stage I–II disease (P = 0.37). By contrast, among patients with TNM stage III–IV disease, those in the high-score group exhibited significantly poorer overall survival compared with those in the low-score group (P = 0.02) (**Supplementary Figure 2A**). In the external validation cohort, a significant survival difference was observed in the TNM stage I–II subgroup, with the high-score group showing inferior survival outcomes (P = 4 × 10^-4^). However, no statistically significant difference was found between the two score groups in patients with TNM stage III–IV disease (P = 0.29) (**Supplementary Figure 2B**).

Subgroup analyses based on histological differentiation further demonstrated the prognostic relevance of the Electrolyte Score. In the training cohort, high Electrolyte Score was significantly associated with worse overall survival in both well-to-moderately differentiated tumors (P = 0.038) and poorly differentiated tumors (P = 0.0023) (**Supplementary Figure 2C**). In the validation cohort, although the difference between the high- and low-score groups was not statistically significant in patients with well-to-moderately differentiated tumors (P = 0.11), patients with poorly differentiated tumors in the high-score group had significantly poorer overall survival than those in the low-score group (P = 0.019) (**Supplementary Figure 2D**). Collectively, these subgroup analyses suggest that the Electrolyte Score showed prognostic stratification ability in several clinically relevant subgroups, particularly among patients with poorly differentiated tumors, although its performance varied across TNM stage and differentiation subgroups.

### Baseline characteristics by electrolyte score groups

The baseline clinicopathological characteristics of patients stratified by the Electrolyte Score are summarized in **Table 2** (training cohort) and **Table 3** (validation cohort). In the training cohort, patients with a high Electrolyte Score were more likely to be male, have advanced TNM stage, and experience a higher event rate than those with a low Electrolyte Score (P = 0.002, P = 0.003, and P < 0.001, respectively), whereas no significant differences were observed in age or histological differentiation between the two groups. In the validation cohort, patients with a high Electrolyte Score were more likely to be younger (P < 0.001), male (P = 0.032), and experienced a higher event rate (P < 0.001) compared to those with a low score. No significant differences were observed in TNM stage or histological differentiation between the two groups.

**Table 2:** Baseline characteristics of gastric cancer patients in the training cohort stratified by electrolyte score (low vs. high).

Characteristics	Low	High	P value
n	543	542	
Age, n (%)			0.211
≥65	225	245	
<65	318	297	
Gender, n (%)			0.002
female	169	124	
male	374	418	
TNM stage, n (%)			0.003
I+II	218	170	
III+IV	325	372	
Differentiation, n (%)			0.882
Moderately-Well	339	336	
Poorly	204	206	
Event, n (%)			< 0.001
alive	259	203	
dead	284	339	

**Table 3:** Baseline characteristics of gastric cancer patients in the validation cohort stratified by electrolyte score (low vs. high).

Characteristics	Low	High	P value
n	172	114	
Age, n (%)			< 0.001
≥65	107	93	
<65	65	21	
Gender, n (%)			0.032
female	51	21	
male	121	93	
TNM stage, n (%)			0.962
I+II	84	56	
III+IV	88	58	
Differentiation, n (%)			0.580
Poorly	120	76	
Moderately-Well	52	38	
Event, n (%)			< 0.001
alive	97	38	
dead	75	76	

### Independent prognostic value of the electrolyte score

**Figure 2:**
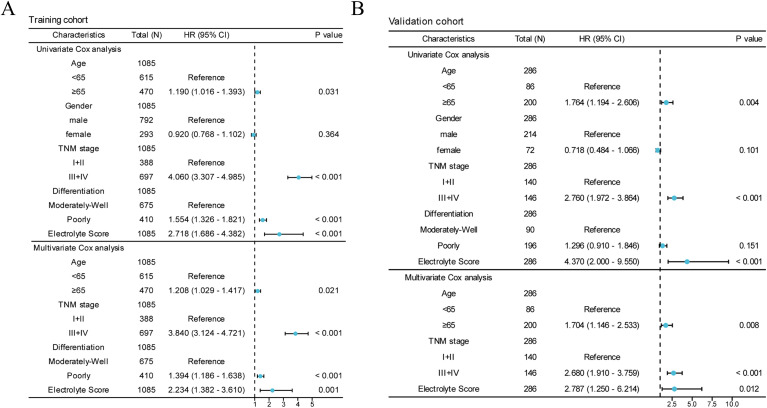
Forest plots of multivariate Cox regression analysis including Electrolyte Score and clinicopathological variables. **(A)** training cohort. **(B)** validation cohort.

The independent prognostic value of the Electrolyte Score was examined using univariate and multivariate Cox regression models incorporating clinicopathological characteristics, including age, gender, TNM stage, and histological differentiation. In the training cohort, multivariate analysis revealed that the Electrolyte Score remained significantly associated with overall survival after adjustment for clinical variables (HR = 2.234, 95% CI: 1.382–3.610, P = 0.001), indicating that it is an independent predictor of prognosis. Age (P = 0.021), TNM stage (P < 0.001), and histological differentiation (P < 0.001) were also independent factors, whereas gender was not statistically significant (**Figure 2A**). Consistently, in the external validation cohort, the Electrolyte Score was independently associated with worse overall survival (HR = 2.787, 95% CI: 1.250–6.214, P = 0.012) (**Figure 2B**), confirming its prognostic value across different patient populations. These results demonstrate that the Electrolyte Score provides prognostic information and could serve as a potential biomarker for risk stratification in gastric cancer.

### Nomogram construction and validation

**Figure 3:**
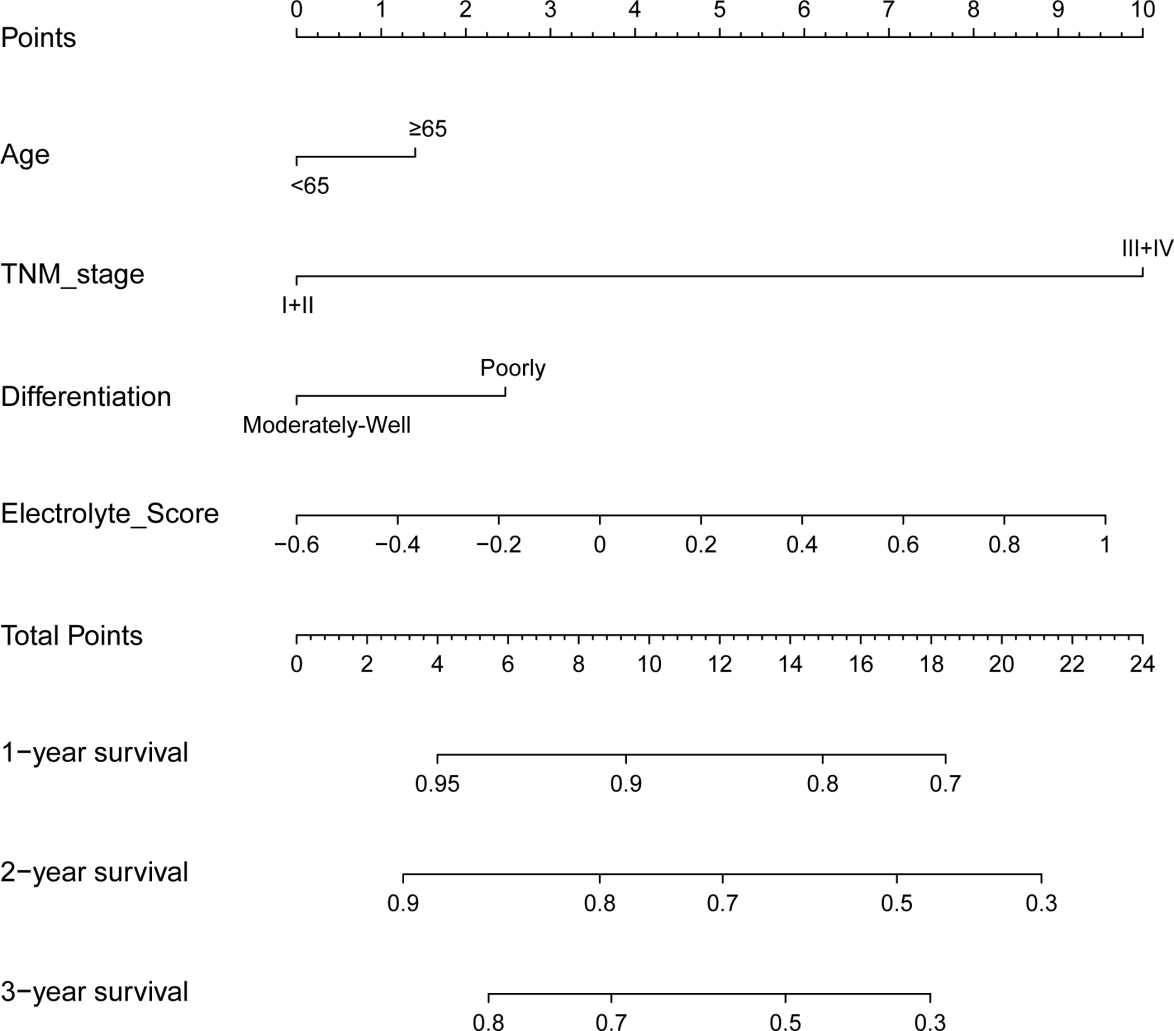
Nomogram integrating the Electrolyte Score, age, TNM stage, and Differentiation to predict 1-, 2-, and 3-year overall survival in patients with gastric cancer.

**Figure 4:**
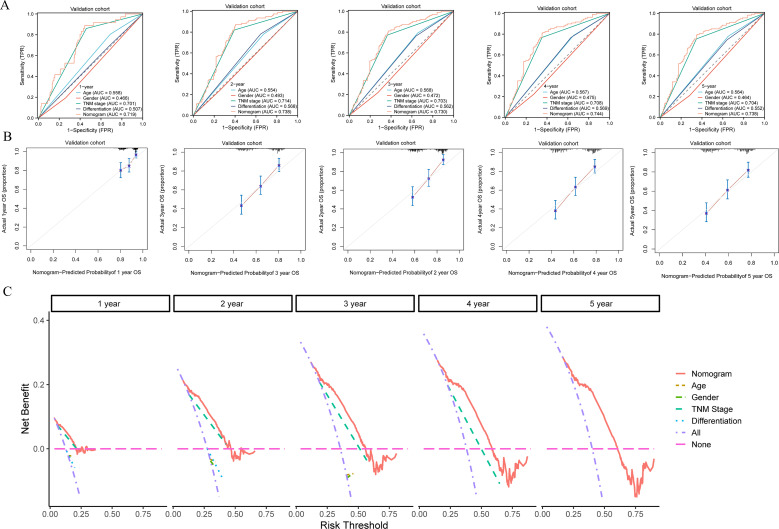
Performance of the nomogram in the training cohort. **(A)** Time-dependent ROC curves for 1-, 2-, 3-, 4-, and 5-year overall survival. **(B)** Calibration curves showing agreement between predicted and observed survival probabilities. **(C)** Decision curve analysis (DCA) demonstrating net benefit relative to individual clinicopathological variables.

Based on the results of multivariate Cox regression in the training cohort, a prognostic nomogram was constructed incorporating independent variables with P < 0.05, including the Electrolyte Score, age, TNM stage, and Differentiation (**Figure 3**). A weighted score was assigned to each variable according to its regression coefficient, allowing individualized prediction of 1-, 2-, 3-, 4-, and 5-year overall survival. The predictive performance of the nomogram was evaluated using time-dependent receiver operating characteristic (ROC) curves. In the training cohort, the AUC values for 1-, 2-, 3-, 4-, and 5-year overall survival were 0.714, 0.743, 0.753, 0.755, and 0.742, respectively, suggesting improved predictive performance compared with models based on individual clinicopathological variables alone (**Figure 4A**). In the training cohort, calibration curves demonstrated good concordance between the survival probabilities predicted by the nomogram and the observed outcomes across all evaluated time points (**Figure 4B**). Moreover, decision curve analysis (DCA) showed that the nomogram provided a greater net clinical benefit than individual variables over a range of threshold probabilities (**Figure 4C**).

**Figure 5:**
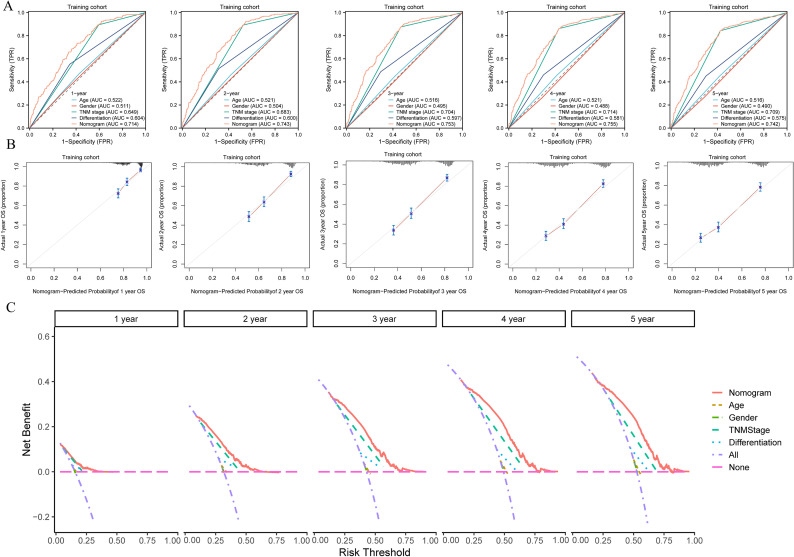
Validation of the nomogram in the external cohort. **(A)** Time-dependent ROC curves for 1-, 2-, 3-, 4-, and 5-year overall survival. **(B)** Calibration curves showing agreement between predicted and observed survival probabilities **(C)** Decision curve analysis (DCA) demonstrating net benefit relative to individual clinicopathological variables.

External validation in the independent cohort further supported the performance and potential applicability of the nomogram. The 1–5 year AUC values were 0.719, 0.738, 0.730, 0.744, 0.738, respectively. calibration curves showed good concordance, and DCA curves similarly demonstrated superior net benefit relative to single clinicopathological factors (**Figures 5A–C**). These results indicate that the nomogram integrating the Electrolyte Score with key clinical variables may provide a useful supplementary tool for individualized prognostic assessment in patients with gastric cancer.

## Discussion

In this multicenter retrospective study, we developed and externally validated an Electrolyte Score based on preoperative serum sodium (Na) and calcium (Ca) levels to predict overall survival in patients with gastric cancer. Our findings suggest that the composite Electrolyte Score can stratify patients into high- and low-risk groups, with higher scores associated with poorer prognosis in both the training and validation cohorts. Although the Electrolyte Score demonstrated significant prognostic value, its predictive performance was further enhanced when combined with key clinicopathological variables in the nomogram.

These results highlight the potential of easily accessible metabolic biomarkers to reflect tumor biology and patient prognosis. Electrolyte disturbances, particularly in Na and Ca, may be linked to alterations in tumor metabolism and the tumor microenvironment, including changes in cellular proliferation, apoptosis, and immune responses (27–44). The use of routine, preoperative laboratory parameters provide a practical and cost-effective approach for risk stratification in clinical settings, complementing traditional clinicopathological factors such as TNM stage and histological differentiation.

The observed prognostic significance of sodium and calcium may reflect their involvement in tumor metabolism and the tumor microenvironment. Sodium plays a key role in maintaining cellular osmotic balance and modulating ion transport, which can influence cancer cell proliferation, migration, and apoptosis. Altered sodium homeostasis has been reported to affect intracellular pH regulation and the activity of sodium-dependent transporters, thereby impacting tumor growth and invasiveness (45–49). Calcium, as a ubiquitous second messenger, is critically involved in signaling pathways that regulate cell cycle progression, apoptosis, and immune responses. Dysregulated calcium signaling in tumor cells can promote proliferation, inhibit apoptosis, and modulate interactions with the tumor microenvironment, including immune cell infiltration and angiogenesis (39, 40, 43). These mechanistic insights provide a plausible explanation for the association between preoperative serum Na and Ca levels and patient survival in gastric cancer. However, as this study was based on circulating serum electrolyte measurements, mechanistic interpretations regarding tumor-cell ion signaling remain speculative and require validation in experimental studies.

Compared with previous studies, our research provides a relatively simple and practical approach to incorporating metabolic biomarkers into prognostic assessment for gastric cancer. While several studies have examined the prognostic relevance of individual electrolytes or other metabolic markers, few have combined multiple preoperative serum electrolytes into a composite score. Our study extends this concept by developing the Electrolyte Score, which integrates sodium and calcium, and validating its performance in an independent external cohort.

From a clinical standpoint, the Electrolyte Score may serve as a practical and low-cost marker for preoperative risk assessment in patients with gastric cancer, given that serum sodium and calcium are routinely included in standard laboratory testing. Nevertheless, its limited discriminatory performance as an independent marker indicates that it should not be used alone for prognostic decision-making. Instead, the Electrolyte Score may be more appropriately interpreted as a complementary metabolic indicator that adds information to established clinicopathological predictors, such as TNM stage, age, and histological differentiation. In the present study, incorporating the Electrolyte Score into a nomogram improved individualized prognostic estimation, suggesting that routinely available metabolic parameters may contribute to refining conventional risk stratification models.

Recent studies have emphasized that the preoperative metabolic evaluation of patients with gastric cancer should not rely solely on individual biochemical indicators, but should also take into account nutritional reserve, systemic inflammation, and skeletal muscle status. Gül et al (50). showed that a reduced Preoperative Cachexia Index, derived from skeletal muscle index, serum albumin level, and neutrophil-to-lymphocyte ratio, was significantly associated with severe postoperative complications, defined as Clavien–Dindo grade III or higher, in patients undergoing gastrectomy for gastric cancer. These findings suggest that metabolic dysfunction before surgery represents a multidimensional process. Within this framework, electrolyte disturbances should not be regarded as isolated laboratory abnormalities, but rather as one manifestation of broader systemic metabolic impairment. Altered sodium and calcium homeostasis may occur together with inflammatory activation, malnutrition, and sarcopenia, collectively compromising physiological reserve and reducing the capacity for postoperative recovery. Therefore, the Electrolyte Score may provide information not only on long-term survival risk, but also on preoperative metabolic vulnerability that may be relevant to surgical outcomes.

In addition, diagnostic timing and access to surgical care may further influence tumor burden and metabolic condition at presentation. Gül and Hafızoğlu reported that the COVID-19 pandemic was associated with delayed diagnosis, stage migration, and impaired nutritional status, including lower body mass index, among patients with gastric cancer (51). Although the cohorts in the present study were collected before the COVID-19 pandemic and were therefore not directly affected by pandemic-related healthcare disruptions, these observations offer important contemporary evidence that delayed diagnosis may contribute to more advanced disease and deterioration of nutritional and metabolic profiles. This context further underscores the clinical importance of accessible preoperative metabolic indicators. As a routinely available and easily calculable biomarker, the Electrolyte Score may complement conventional clinicopathological factors by capturing aspects of preoperative metabolic vulnerability.

Several limitations should be considered when interpreting the findings of this study. First, because of its retrospective nature, selection bias could not be completely avoided. Although only a small proportion of patients were excluded due to incomplete data, the baseline imbalance between the training and validation cohorts may still restrict the generalizability of our results. Second, despite the use of consistent reporting units for electrolyte measurements, differences in laboratory procedures, assay platforms, and reference ranges across centers may have contributed to measurement variability. Third, the Electrolyte Score showed limited predictive accuracy when used alone, as indicated by the relatively low time-dependent AUC values. Therefore, it should not be regarded as an independent standalone tool for clinical decision-making, but rather as a complementary biomarker to be interpreted together with established clinicopathological factors. Fourth, the relatively small size of the external validation cohort may have reduced the stability of the validation results, especially in subgroup analyses. Fifth, the median Electrolyte Score was adopted as the cut-off value because no clinically established threshold is currently available. Although this strategy is straightforward and reproducible, future studies are required to determine whether optimized or clinically meaningful cut-off values can further improve risk stratification. Sixth, serum calcium may be influenced by albumin concentration and nutritional status, whereas serum sodium may be affected by hydration status, renal function, medication use, and perioperative management. These potential confounding factors could not be fully controlled in the present retrospective analysis. Finally, prospective multicenter studies incorporating standardized laboratory protocols, longitudinal electrolyte measurements, and comprehensive nutritional and inflammatory assessments are needed to further validate and refine the clinical applicability of the Electrolyte Score.

In conclusion, the Electrolyte Score derived from preoperative sodium and calcium levels provided independent prognostic information for patients with gastric cancer, although its discriminative ability was limited when used alone. Its incorporation into a nomogram together with clinicopathological variables offered a more comprehensive approach for individualized prognostic assessment. Future prospective studies with larger cohorts are needed to further validate and refine this model. In addition, repeated electrolyte measurements and the integration of other metabolic or microenvironment-related biomarkers may improve predictive performance and further clarify the biological basis underlying its prognostic relevance.

## Data Availability

The raw data supporting the conclusions of this article will be made available by the authors, without undue reservation.

## Supplementary material

Supplementary Figure 1 Predictive value of the Electrolyte Score for gastric cancer. **(A, B)** Time-dependent receiver operating characteristic (ROC) curves of the Electrolyte Score for predicting 1-, 3-, and 5-year overall survival in the training **(A)** and validation **(B)** cohorts. **(C, D)** Kaplan–Meier survival curves of patients stratified into high- and low-risk groups based on the median Electrolyte Score in the training **(C)** and validation **(D)** cohorts. P-values were calculated using the log-rank test.

Supplementary Figure 2 Subgroup Kaplan–Meier survival analyses of the Electrolyte Score according to TNM stage and histological differentiation. **(A)** Subgroup survival analysis according to TNM stage in the training cohort. **(B)** Subgroup survival analysis according to TNM stage in the validation cohort. **(C)** Subgroup survival analysis according to histological differentiation in the training cohort. **(D)** Subgroup survival analysis according to histological differentiation in the validation cohort.
