# Supplementary figures and images for: Preoperative electrolyte-based metabolic biomarkers for prognostic assessment in gastric cancer: a multicenter study
Source: Front Endocrinol (Lausanne). 2026 Jun 11;17:1852688. doi: 10.3389/fendo.2026.1852688 (PMC13293866; doi:10.3389/fendo.2026.1852688)

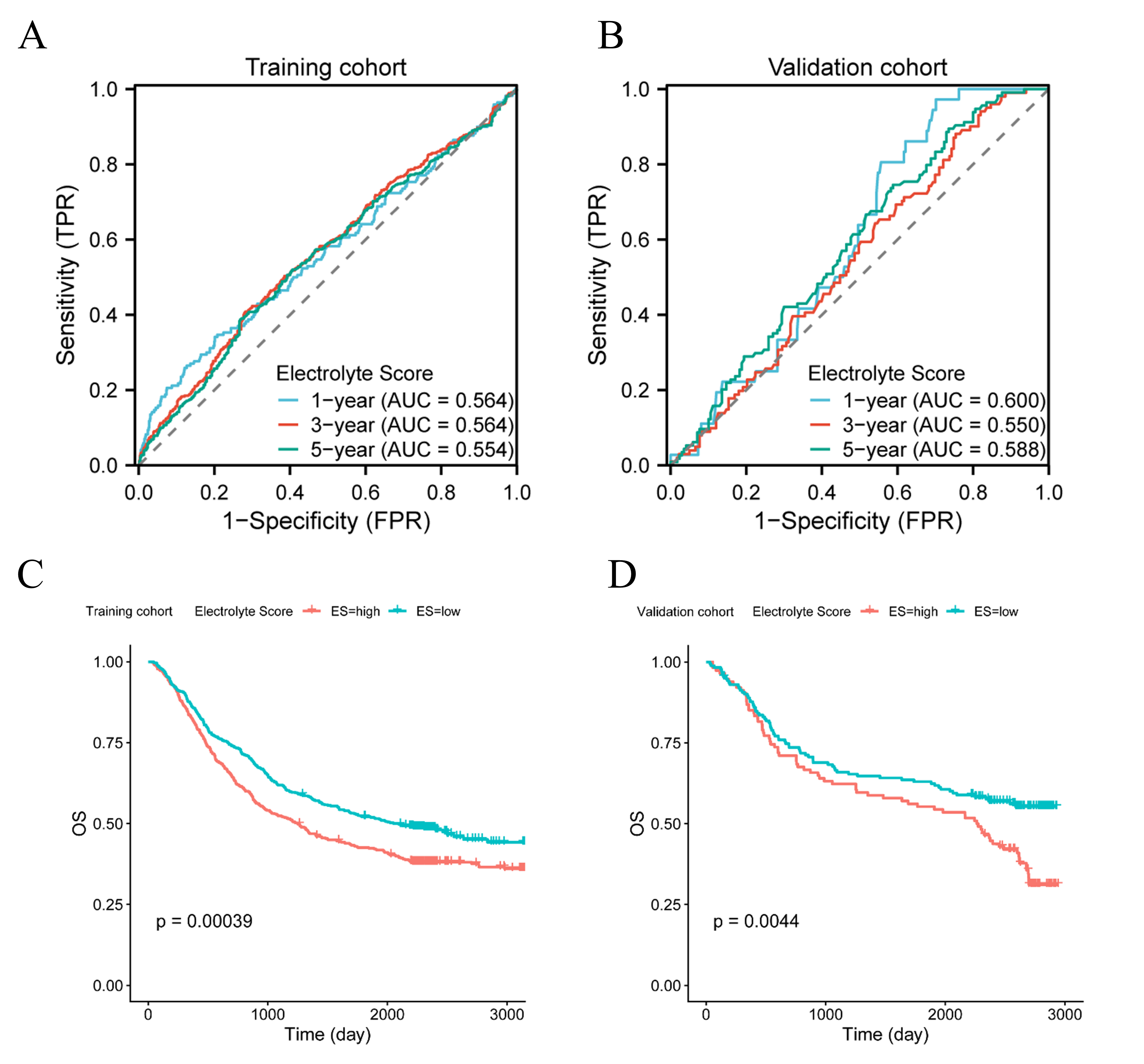

Supplement: Supplementary Figure 1 — Predictive value of the Electrolyte Score for gastric cancer. (A, B) Time-dependent receiver operating characteristic (ROC) curves of the Electrolyte Score for predicting 1-, 3-, and 5-year overall survival in the training (A) and validation (B) cohorts. (C, D) Kaplan–Meier survival curves of patients stratified into high- and low-risk groups based on the median Electrolyte Score in the training (C) and validation (D) cohorts. P-values were calculated using the log-rank test. [file Image1.tif]

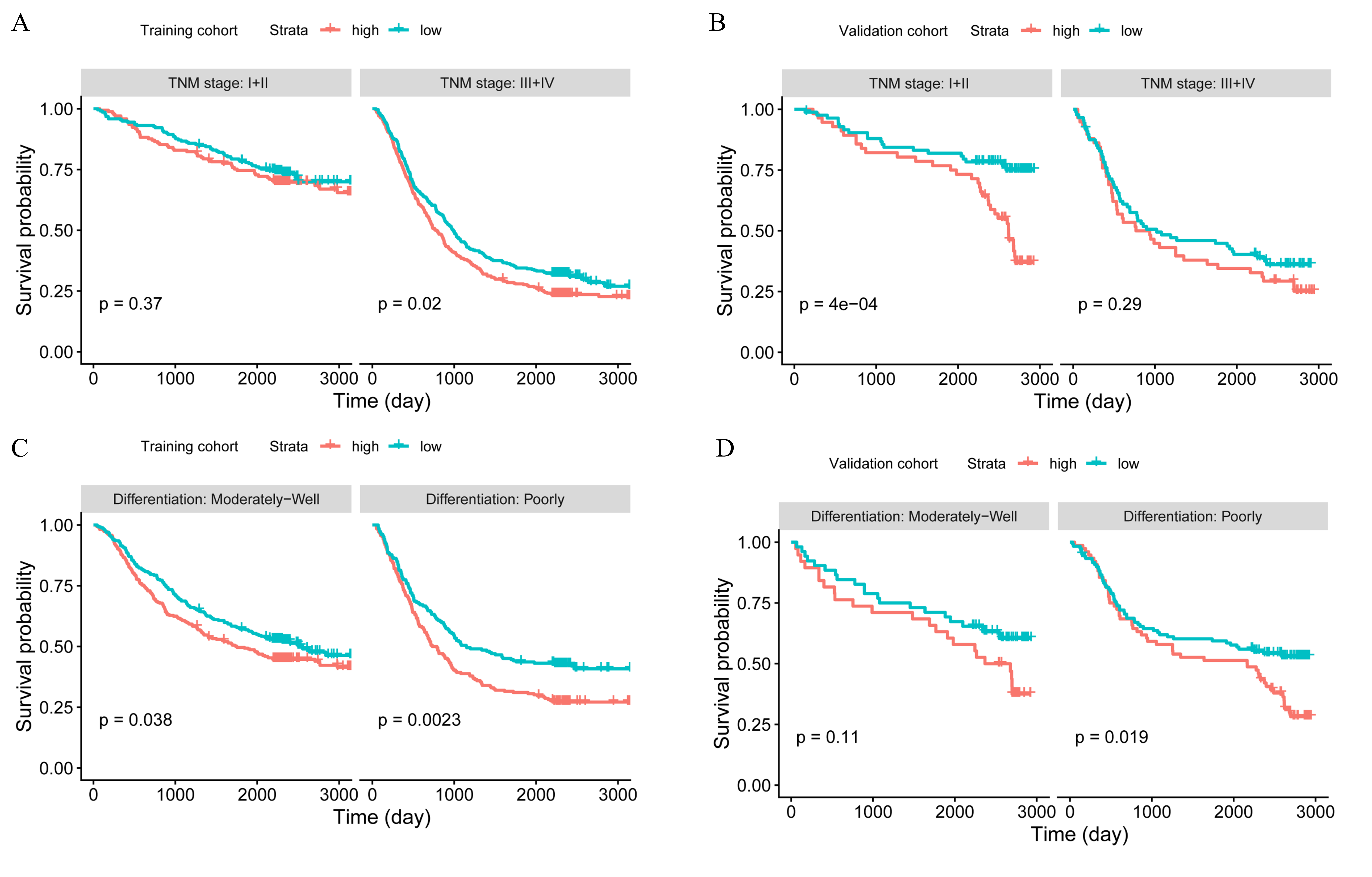

Supplement: Supplementary Figure 2 — Subgroup Kaplan–Meier survival analyses of the Electrolyte Score according to TNM stage and histological differentiation. (A) Subgroup survival analysis according to TNM stage in the training cohort. (B) Subgroup survival analysis according to TNM stage in the validation cohort. (C) Subgroup survival analysis according to histological differentiation in the training cohort. (D) Subgroup survival analysis according to histological differentiation in the validation cohort. [file Image2.tif]
